# To what extent does surrounding landscape explain stand-level occurrence of conservation-relevant species in fragmented boreal and hemi-boreal forest? – a systematic review

**DOI:** 10.1186/s13750-024-00346-1

**Published:** 2024-08-12

**Authors:** Malin Undin, Anita Atrena, Fredrik Carlsson, Mattias Edman, Bengt Gunnar Jonsson, Jennie Sandström

**Affiliations:** 1 Department of Natural Sciences, Design, and Sustainable Development, Mid Sweden University, Sundsvall, SE-851 70 Sweden; 2 Department of Wildlife, Fish and Environmental Studies, Swedish University of Agricultural Sciences, Umeå, SE-901 83 Sweden

**Keywords:** Abundance, Biodiversity, Deadwood-dependent species, Fragmentation intensity, Habitat loss, Indicator species, Isolation, Red-listed species, Species richness, Taiga

## Abstract

**Background:**

Forestry and land-use change are leading causes of habitat loss, degradation, and fragmentation worldwide. The boreal forest biome is no exception, and only a small proportion of this forest type remains intact. Since forestry will remain a major land-use in this region, measures must be taken to ensure forest dependent biodiversity. Stand level features and structures promoting conservation relevant species have received much attention, but the landscape level perspective is often missing. Hence, we review the literature that has related fragmentation in the surrounding landscape to occurrence of threatened, declining, red-listed, rare, or deadwood dependent species as well as those considered to be indicator, flagship, umbrella, and/or keystone species in a given boreal forest stand.

**Methods:**

A comprehensive search string was developed, benchmarked, and adapted for four bibliographic databases, two search engines, and 37 specialist websites. The online evidence synthesis tool Cadima was used for screening of both abstracts and full texts. All articles meeting the inclusion criteria were subject to study validity assessment and included in a narrative table. Studies reporting means and variance were included in quantitative meta-analysis when more than 3 comparable studies were available.

**Results:**

The searches resulted in 20 890 unique articles that were reduced to 172 studies from 153 articles. These studies related stand level presence, abundance, species richness, and/or composition of conservation relevant species to landscape factors such as: categorical fragmentation intensity (higher vs. lower), amount of habitat or non-habitat, distance to habitat, and/or habitat configuration, on scales ranging from tens to tens of thousands of ha. Forty-three studies were suitable for meta-analysis. These showed a significant negative effect of fragmentation on both presence and abundance of conservation relevant species, as well as a near significant trend for species richness. This was particularly clear when fragmentation was measured as distance to surrounding habitat for presence, and as habitat amount for abundance. The organism groups with the strongest support for a negative effect of fragmentation were wood fungi and birds.

**Conclusion:**

As hypothesised, there is strong support for negative effects of fragmentation in boreal forest. These results emphasize the negative consequences of the intensive forestry and associated landscape transformation that has been the norm for the last century. We argue that this should have direct implications for policy makers to shift towards including a landscape perspective in all planning of harvesting, preserving, and restoring forest. In addition, we found that research effort has been very uneven between organism groups, that studies on landscape change over time were rare, and that many studies have not quantified the difference in fragmentation intensity among landscapes making it difficult to quantify the extent of the negative effect. One way forward would be to revisit the studies included here in to incorporate change over time, as well as a true quantification of landscape fragmentation. By doing so, the scale of the negative effects would be much better analysed, which would greatly assist conservation practitioners all throughout the boreal forest biome.

**Supplementary Information:**

The online version contains supplementary material available at 10.1186/s13750-024-00346-1.

## Background

Forestry and land-use change are leading causes of habitat loss worldwide, with negative consequences for many forest species [1, 2]. In addition to habitat loss per se, forest harvesting tends to fragment the remaining forest into smaller, isolated units (hereafter referred to as stands; [3–5]). Effects of habitat loss and fragmentation can be difficult to separate [9, 10], yet a growing body of evidence supports that fragmentation of continuous habitat have direct consequences for many conservation-relevant species, including birds, mammals, beetles, fungi, and lichen ([6, 9, 11–14], but see [8]). Fragmentation can, for instance, accentuate, change, or even alleviate the effects of habitat loss [6–8]. Furthermore, modelling suggests that climate change can further aggravate forest fragmentation effects [15]. In turn, fragmentation, may result in edge effects that change local climate, temperature, and wind conditions, potentially causing further stress to already declining populations [12, 16–18].

A common approach in studies of stand level diversity is to apply a landscape perspective in a biogeographical context that account for stand size and isolation, and that effectively treat fragments as islands [19–21]. Thus, the most well studied consequences of fragmentation are poor recruitment, reduced (functional) connectivity, and associated loss of geneflow caused by long distances between habitat patches [3, 12, 22]. These studies, for instance, suggest that species with lower dispersal ability and a shorter lifespan should be more sensitive to fragmentation [23], and/or that different organism groups and species will be affected by fragmentation at different spatial and temporal scales [6, 18, 24]. However, compared to actual islands, forest stands will fall along a gradient from effectively continuous populations, to functioning meta-populations with a balance of extinction and (re)colonization, to non-viable meta-populations where sub-populations lose connectivity and slowly disappear [25, 26]. The configuration, composition, and history of the surrounding landscape (or matrix) will directly affect where along this gradient a stand sits [18, 27–29].

The theoretical importance of spatial and temporal changes to both landscape context and configuration is well understood [7, 9, 30–33]. Empirical studies have also found effect of landscape composition and configuration of several organism groups [6, 13]. However, reviews of such studies have found that the relationship is complex and depend on habitat amount remaining [6] or landscape size investigated [13]. Despite this, the effect of landscape-level variables on stand-level diversity remains underrecognized and their effect require additional focus [12, 18, 34–39]. For instance, indicators of high conservation value in forest usually focus on stand level features such as stand size, structure, and deadwood amount [40], and do not consider the surrounding landscape. In line with this, some reviews addressing forest fragmentation and diversity leave out the landscape aspect entirely [41, 42].

The boreal biome contains about 27% of all forest globally [4, 43, 44]. Only about 8.5% of the this forest is formally protected [44], which falls way short of the new global targets of protecting at least 30% of all terrestrial areas by 2030 in a representative and functional network [45]. The boreal forest has been intensively used for forestry and subject to land use change, causing a high level of habitat loss and fragmentation [44, 46, 47]. As a result, only 11% of the boreal forest remains relatively intact, and the average size of a continuous boreal forest is approximately 336 ha [33]. In addition, human impact has been noticeably uneven, resulting in most remaining intact boreal forest being found in Russia and Canada [44, 47]. By contrast, in Fennoscandia, almost 1% of the standing forest has been clear cut annually since the onset of large-scale rotation forestry in the 1950s [4, 46, 48, 49]. In addition, a substantial proportion of the remaining forest has been affected by thinning and/or small-scale harvesting [4, 46, 50]. Given the critical value of the remaining natural boreal forest, the continued forest harvesting and land use change, and the importance of dispersal and landscape permeability for species diversity, it is logical to assume a substantial effect of fragmentation on both incidence and abundance of conservation-relevant species in boreal forest stands. Furthermore, the slow turn over, combined with that many boreal forest species are highly specialised and recognised as threatened [51], suggests high risk for extinction debt in this zone [13, 23, 24, 38]. Lastly, the boreal zone has large landscape variation due to differences in historical land-use and level of human impact [3, 46, 47, 52], suggesting a high availability of comparisons to study. At the same time, boreal forest contains multiple forest types, with their associated species pools, suggesting potential for different responses to the same type or level of fragmentation.

Taken together, there is an urgent need to protect remaining natural boreal forests and the species they harbour, to introduce sustainable forest management, and identify areas with potential for restoration [53]. For this, involved stakeholders need better understand the premises for conservation success in fragmented landscapes, and particularly the role of the matrix, in order to interpret observed diversity declines; prioritise among interventions, stands, and sites to protect and restore; and plan for green infrastructure (i.e., reconstructed connectivity) in boreal and hemi-boreal forest. In addition, it is well established that deadwood-dependent species are vulnerable to lost connectivity and substrata continuity and thus that they are good indicators of pristine like forest [13, 24], but complementary perspectives across species groups are needed.

Thus, we address the review question: ‘To what extent does surrounding landscape explain stand-level occurrence of conservation-relevant species in fragmented boreal and hemi-boreal forest?” Conservation-relevant species, is defined herein as any threatened, declining, red-listed, rare, or deadwood dependent species as well as those considered to be indicator, flagship, umbrella, or key-stone species.

### Stakeholder engagement

During the development of this review, two consultation meeting were held between the review authors and an advisory group consisting of representatives from several key Swedish agencies involved in forestry and forest protection; namely, the Swedish Environmental Protection Agency (SEPA; Naturvårdsverket), the Swedish Forest Agency (Skogsstyrelsen), the County Administrative Boards (Länsstyrelserna) in Västernorrland, Jämtland, Norrbotten, and Gävleborg, and three of the main forestry companies in Sweden: Sveaskog, Holmen Skog, and SCA.

The main objectives of the first meeting were to discuss the importance of considering the surrounding landscape in conservation planning, identify knowledge gaps and landscape parameters, as well as other effect modifiers of interest for the stakeholders. The main objective of the second meeting was to discuss the results, their presentation, their contribution to conservation management, and the knowledge gaps they identify.

The main outcome of these meetings was that the stakeholders showed a great interest in the subject. Specifically, there interests lie in utilising the review and included articles then prioritising what and where to protect and restore, to compare areas, and to efficiently contribute to so called green infrastructure. The meetings directly affected how we defined our primary and secondary questions, PECO, search terms, eligibility criteria, effect modifiers of interest, and data parameters to extract. Specific examples were that the stakeholders convinced us to not use any size limitations for what counted as landscape, and stressed the practical importance of breaking down results by forest type. Furthermore, the meetings directly impacted the final analyses, presentation, and discussion of the results. Specific examples are that the stakeholders stressed three knowledge gaps important for them: identifying threshold values for amount of remaining habitat in the landscape, evaluate the difference in impact between different matrix types, and better evaluate the effect of change over time (see additional file 1 for more details).

## Objectives

The main objective of this review is to assess the primary question: ‘to what extent does surrounding landscape explain stand-level occurrence of conservation-relevant species in fragmented boreal and hemi-boreal forest?’ This was broken down into the following population, exposure, comparator, outcome (PECO) components:

### Population

Boreal and hemi-boreal forest, defined as any forest within the boreal zone and the hemi-boreal transition zone which cover all or parts of the following countries: Canada, Scotland, Iceland, Norway, Sweden, Finland, Estonia, Latvia, Lithuania, Belarus, Russia, Mongolia, Japan, and the American states Alaska, Maine, and Minnesota [44, 54–57]. Hereafter the term boreal forest is used to mean boreal and hemi-boreal forest for simplicity.

### Exposure

Fragmentation and habitat loss, defined as the breaking apart of larger forest tracts into smaller forest stands surrounded by a matrix directly affected by forest harvesting and/or other types of land-use change.

### Comparator

Stands in landscapes that differ in terms of fragmentation intensity, habitat amount, stand isolation, and/or habitat configuration.

### Outcomes

Occurrence, i.e. presence, abundance, richness, or composition, of conservation-relevant species. Conservation-relevant is defined as rare, threatened, red-listed, area sensitive, old-growth forest or dead wood dependent, indicator, keystone, flagship, or umbrella species.

**Fig. 1:**
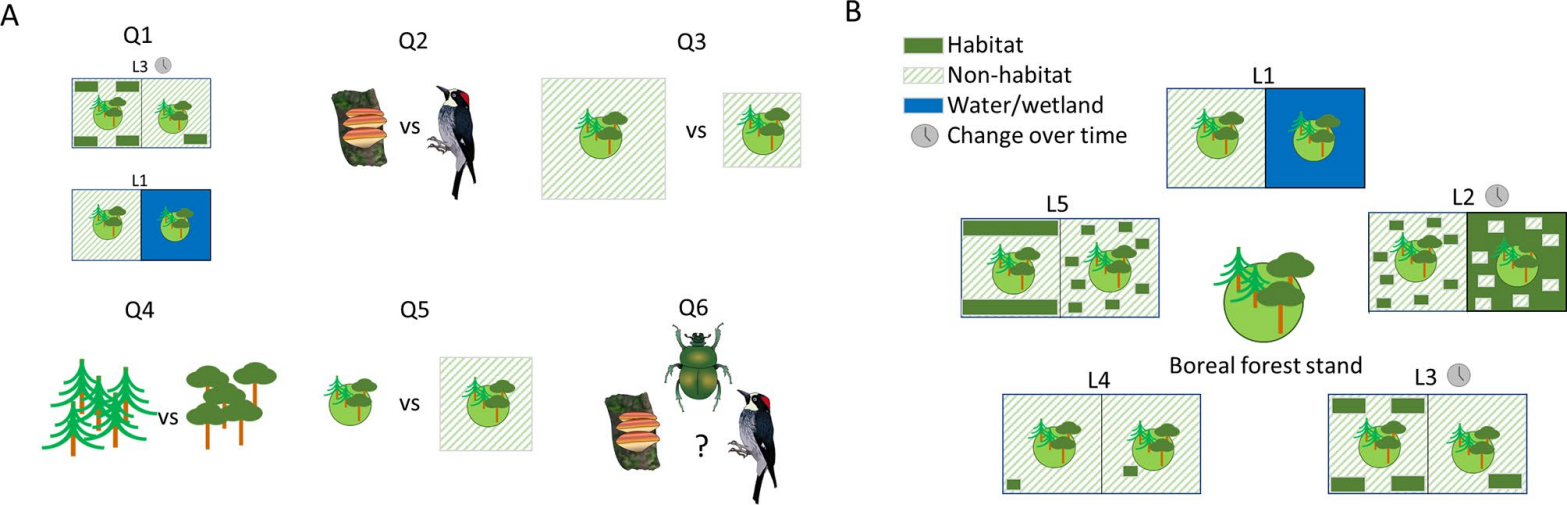
Conceptual illustration of our hypotheses (**A**) and the landscape factors considered in this review (**B**). We hypothesised that fragmentation of the surrounding landscape has a negative effect on occurrence of conservation relevant species in a given stand. Furthermore, we hypothesised that the extent of this relationship will depend on the landscape factor (Q1), organism group (Q2), size of the landscape studied (Q3), the forest type (Q4), as well as stand-level factors (Q5). Lastly, we hypothesised that the evidence base and hence the knowledge gaps will vary between organism groups (Q6). The landscape factors considered are: L1 Human vs. Natural fragmentation; L2 Fragmentation intensity; L3 Amount of habitat, non-habitat of its change over time; L4 Distance to habitat or non-habitat; L5 Habitat configuration (spatial distribution of habitat)

We hypothesised that there is a negative effect of fragmentation in the surrounding landscape on occurrence of conservation-relevant species. Furthermore, we identified a number of sub hypothesis or secondary questions related to how the landscape effect is manifested and depend on a range of factors. The extent of the effect may differ among organism groups and, for instance, be higher for groups with poor dispersal ability [23]. The extent of the effect may also depend on the exact aspect of the landscape analysed, i.e. the explanatory power will be higher if a variable more important to the organism has been quantified [6]. We hypothesised a bell-shaped relationship between the explanatory power and the size of landscape studied, i.e. quantifying fragmentation on a too small or a too large area will have a lower explanatory power for the occurrence of conservation relevant species [6, 19, 24]. The extent of the impact of the surrounding landscape may also be affected by parameters within the stand, such as its forest type. Lastly, we hypothesised that the evidence base and hence the knowledge gaps will vary between organism groups (Fig. 1).

## Methods

The review process followed the standards and guidelines from the Collaboration for Environmental Evidence (CEE; 58), the reporting standards from ROSES (Additional file 2), and our published protocol (59; http://www.proceedevidence.info nr PROCEED-22-00027), except when indicated below.

### Deviations from protocol

All deviations from the published protocol were minor and done to accommodate for the relatively high number of studies identified, their heterogeneity, and their exact content. The largest change was that the search string was edited for Web of Science to only include articles from relevant Web of Science Categories, namely: Ecology, Environmental Science, Forestry, Biodiversity Conservation, Zoology, Ornithology, Microbiology, Entomology, Biology, Mycology, and Parasitology. With this adjustment, all 43 benchmark articles were still included in the Web of Science search results, and a pilot screening confirmed that potentially relevant articles excluded in the Web of Science search were included in the SCOPUS search. No such changes were done for the other databases used. Due to the very large number of species included in the studies, it was also deemed unfeasible to provide a table of all studied species. The distribution of topics in the studies led to slight adjustments of the secondary questions, hypothesis, and categories of landscape factors. Clarification was added to the study validity criteria. The data extraction sheet was revised based on the content of the identified studies and somewhat simplified due to the large number of studies and their heterogeneity. The large geographic range of studied landscapes in many studies made it meaningless to extract coordinates, and country was deemed as the sufficient level of geographic information. Adjustments were also done to the meta-analyses (see below).

### Search strategy and search string

The search for articles relied on a predefined list of four bibliographic databases, two search engines and 37 specialist websites [59] and supplementary searches through reference lists of previously published relevant reviews. Articles with abstracts written in English, Swedish and Norwegian were read but only articles written in English made it through the screening, and no translation assistants was needed. The initial search was done February 1st 2022, with complementary searches done in March and August 2022, and an annual search update was done March 7th 2023.

The search string and search blocks were as in the published protocol [59]. In short, the first block defined the relevant population and thus includes terms such as ‘forest*’, ‘wood*’, ‘deadwood’, ‘boreal’ and ‘hemi-boreal’ as well as the names of the relevant countries and regions that host these bioregions (Table 1; Additional file 2). The two following blocks defined the relevant exposure and comparators i.e. articles with a landscape component, and studies of forest fragmentation. The fourth block defined the relevant outcomes, specifically the relevant units of measure for the occurrence of conservation-relevant species. The comprehensiveness of the search was assured with a benchmark list of 43 articles as per the published protocol. Each block was combined with the Boolean operator “AND”. Slight differences were applied to the different databases, namely: the search targeted “All fields” in PubMed and CABI; title, abstract, and keywords in SCOPUS (“TITLE-ABS-KEY”), while in Web of Science, all fields (“ALL”) was used for the first block and topic (“TS”) for the other three, which covers title, abstract, and keywords added by the author and by Web of Sciences’ own algorithm that searches for key phrases in the reference list. Restrictions based on categorisation of articles were only used for Web of Science (see above).

**Table 1:** Final search string with four blocks formatted for web of Science Core Collection

Block	Terms
1. Population	ALL = ((forest* OR wood* OR deadwood* OR dead-wood*) AND (boreal* OR boreonemoral OR hemiboreal OR hemi-boreal OR taiga OR Sweden OR Finland OR Fennoscandia OR Norway OR Canada OR Alaska OR Estonia OR Russia OR Scotland OR Iceland OR Mongolia OR Japan OR Siberia OR Latvia OR Lithuania OR Maine OR Minnesota OR Belarus)) AND
2. Exposure/Comparator – Landscape scale	TS = (landscape* OR region* OR spatial OR provinc* OR “large-scale” OR surrounding OR fragment* OR matrix) AND
3. Exposure/Comparator – Fragmentation	TS = (fragment* OR continu* OR connectivity OR isolate* OR “habitat loss” OR woodlot* OR “forest stand*” OR metapopulation OR “habitat patch*” OR configuration OR “old-growth forest*” OR “woodland key habitat*” OR “management histor*” OR “land-use histor*” OR “land use histor*” OR “historic* land use”) AND
4. Outcomes	TS = (biodiversity OR “species richness” OR distribution OR abundan* OR occurrence OR composition OR extinction* OR diversity OR densit* OR cover OR coloni*ation* OR occupancy OR dispersal OR community OR viab* OR “population trend*” OR activity OR “species turnover” OR nesting OR incidence OR “genetic diversity” OR “genetic structur*” OR “isolation by distance” OR “isolation-by-distance”)

### Article screening and study eligibility criteria

The online evidence synthesis tool Cadima (version 2.2.4.2; [60]) was used to screen the identified articles. As specified in the protocol [59] screening was conducted as a two-step process; based on title and abstract, and on the full text, respectively. To confirm consistency among authors, the built-in consistency check in Cadima was used. During the consistency checks, four authors independently screened 100 titles and abstract, and 20 full text articles, per iteration. This was repeated until a kappa value > 0.6 was reached [61]. The same four authors then conducted the screening, but all authors were involved in the discussions of any inconsistencies around the interpretation of the inclusion criteria or the content of the articles. After the consistency check, there was also a 5% and a 10% overlap between authors in the screening of the abstracts and full text respectively.

### Eligibility criteria and reasons for exclusion

No changes were made to the eligibility criteria or definitions compared to the published protocol [59]. In short, the *relevant population* was defined as studies conducted in boreal hemi-boreal (also sometimes referred as boreonemoral) forest [44, 54–57] in the countries and regions defined above (see “Objectives”). Studies were excluded if only part of the study was conducted in boreal or hemi-boreal forest and outcomes from boreal and hemi-boreal forest could not be separated from other results. *Relevant exposure* was defined as studies where the surrounding forest landscape had been fragmented through direct human impact, i.e., through the felling of trees for forestry or any form of land-use change and included only studies addressing direct effects of fragmentation at landscape scale (hence studies of edge effects were excluded). *Relevant comparators and types of studies* was defined as primary studies that had compared two or more landscapes and looked at how landscape factors affected the occurrence of conservation-relevant species on the level of a defined stand. Any factor describing the area (of any size) surrounding the stand (i.e. the matrix) was deemed a landscape factor. Based on results of the screening, the categorisation of explored landscape factors were altered compared to protocol; the final categories were: L1 Human vs Natural fragmentation; L2 Categorical fragmentation intensity; L3 Amount of habitat, non-habitat, or its change over time; L4 Distance to habitat or non-habitat; L5 Habitat configuration (spatial distribution of habitat; Fig. 1B). Habitat and non-habitat were defined based on the study authors assumption of a positive or negative effect of a higher amount in the surrounding landscape. Habitat was usually forest, either all or forest specified by age, species composition, or status (such as “pristine like”). Non-habitat was most commonly clear-cuts or farmland. Managed forest of different ages could be defined as habitat or non-habitat depending on the study authors’ assumption.

*Relevant outcomes* were defined as the presence, abundance, species richness, and/or composition of conservation-relevant species of all organism groups. Conservation-relevance (defined above under “Objectives”) was specified by the authors of the original study. The specification had to relate to the species studied, general statements about conservation-relevance of an entire species group were not considered sufficient. Studies were excluded if they looked at a large number of species, some of which the study authors stated as conservation relevant, if the outcomes were not presented separately for the conservation relevant species.

Each article could be deemed to include one or more eligible studies to be included in the review. All included studies were assessed for independence. Studies were considered independent if they used non-overlapping study plots and separate analyses. Additionally, studies using fully or partly overlapping study plots could be considered independent if they analysed (i) different landscape variables, (ii) different measurements of occurrence, such as presence vs. abundance and/or (iii) different organism groups in separate statistical analyses. Examples of i could be one study comparing “high” vs. “low” fragmentation, a second using a subset of the same plots to compare amount of habitat in the landscape, and a third returning later to the same plots to study change over time. Studies utilizing partly or fully overlapping study plots were considered belonging to the same project. Studies were also considered to belong to the same project if study plots were separate but the methodology, organism group and (some of) the authors overlapped.

### Study validity assessment

All studies passing full text screening were subject to study validity assessment (also referred to as critical appraisal) to categorise the studies as being of low, medium, or high risk of bias based on study design and/or methodology. The criteria were chosen based on previous review protocols published in EE, on the critical appraisal tool developed by CEE (Version 0.3), and a pilot assessment utilising seven of the benchmark articles. Some adjustments and clarification of the study validity criteria was done compared to the published protocol (Additional file 3; 59). Each study was assessed based on 10 criteria: (1) were landscapes, stands and/or plots comparable?; (2) were potential confounders accounted for?; (3) were study sites selected in a bias free way?; (4) were stand and landscape variables appropriately quantified and described?; (5) was the sample size sufficient?; (6) was pseudo replication avoided and/or accounted for?; 7–9) was the outcome quantified, the survey method, and the statistical method appropriate for the study?; and 10) were the results sufficiently reported? If the answer to all criteria was Yes or if any Nos were accounted for, the study was considered of low risk of bias; if the answer was Potentially no, Partly no, or Partly unclear, for at least one criteria, the study was considered of medium risk of bias; if the answer was No or too little information was provided to give an answer, the study was considered of high risk of bias. For more details on criteria, and the results of the assessment for each study see Additional file 3. Three authors conducted the study validity assessment. Seven articles were used to ensure consistency among these authors (the same seven articles used for the pilot study of the data extraction; 59), in addition, the assessment of many studies was conducted by discussion among the authors. The assessment results were considered in analyses (see below) and in phrasing of the narrative review, and are discussed below, but no study was excluded based on the assessment.

### Data extraction, synthesis, and presentation

Based on the number of relevant studies and their content, data extraction was not conducted in Cadima, and the data extraction sheet, coding options, and effect modifiers were modified compared to the published protocol (Additional file 4). Extraction was based on the articles and published supplementary material, and no contact with study authors to request clarification and/or data was considered needed. When one of the review authors was an author of an identified article, they were not involved in the screening, appraisal, data extraction, or narrative synthesis of that article.

All studies passing the full text screening were included in the narrative synthesis and in the attached narrative synthesis table (Additional file 4). The written summary presented herein, only discuss combinations of organism group, outcome, landscape factor, landscape size with a sufficient and manageable number of studies. This to reduce risk of unfair compilation of the data, while keeping the text relatively short. Further information about all included studies can be found in the attached table (Additional file 4).

### Quantitative synthesis and Meta-analyses

For 43 of the included studies, means, samples sizes, and variance could be extracted from tables or figures, to calculate effect sizes as Hedge’s g. These were included in quantitative meta-analyses. The heterogeneity among studies in how fragmentation was defined and quantified meant that no biologically relevant continuous variable could be extracted from all studies. Hence, no meta-regression was attempted. In addition, too few studies presented correlation data, and thus no meta-regression of correlations was conducted. The online image analysing tool WebPlotDigitizer (version 4.5) was used when needed to extract numerical values from figures. Variance was extracted as standard deviation when possible, when other units of variance were reported, these were recalculated to standard deviation. Most of these studies presented either a mean landscape parameter (such as amount of habitat) around occupied versus unoccupied stands, or a mean abundance or species richness in more versus in less fragmented landscapes. Thus, each study was treated to generate a two levelled, categorical comparison (occupied vs. unoccupied or higher vs. lower fragmentation intensity) which resulted in three meta-analyses of the effect of fragmentation: one on presence, one on abundance, and one on richness of conservation relevant species.

When results from multiple years were presented, the most recent was used. When results were presented for multiple landscape sizes, a 500 m radius, or the option closest was used, since this was the median size across included studies and thus deemed most comparable. When multiple variables had been quantified as habitat and non-habitat, one was chosen. For non-habitat, clear-cuts were used when possible since this was the most common variable and the one most closely linked to our review question. For habitat, the variable most clearly defined as suitable for the species by the study authors was used, i.e. if a species was described as requiring old forest, but habitat had been quantified both as all forest and amount of old forest, amount of old forest was used in the analyses. When results were presented separately for several species, the average across all conservation relevant species was calculated and used. However, results from different organism groups (such as wood fungi and saproxylic insects) were analysed separately.

When study authors had divided stands in more than two categories, these were combined to generate a “high” vs. a “low” fragmentation intensity. When one stand type clearly represented a control, this was used as the “low” and remaining categories were combined to represent “high” fragmentation intensity. If categories were clearly grouped in two by the study authors, these groups were used to represent “high” and “low”, and results were combined within each group. If stands and/or landscapes represented a gradient (most commonly a geographic gradient), the split between high and low was defined by the median, i.e. for a gradient with five levels from less to more fragmented, categories one and two were combined and categories four and five were combined. When a scatterplot was presented, each outcome value was extracted and split into two groups representing “high” and “low” fragmentation. This was done using the median (such as the median amount of habitat in the landscape) excluding the median value (or the two values in the middle when an even number of data points was presented). The mean and standard deviation was then calculated for each group. When groups where combined, the standard deviation for all groups were decomposed and recombined using the Cochrane’s Equation [62].

For studies of presence/absence, we used the value for landscapes around occupied *versus* around unoccupied stands when this was reported. However, some studies compared the landscape around occupied stands *versus* around a random (not surveyed) plot in the landscape and then these data were used instead. When intermittent presence categories were included in the study (such a “presence sometimes”, or “sited but breeding not confirmed”) these were excluded from the meta-analyses. For the meta-analyses, all studies of presence/absence were aligned so that a negative effect size meant a negative effect of fragmentation. Hence, for studies comparing the amount of habitat, the occupied sites were used as the baseline, the hypothesis being that occupied stands are surrounded by *more* habitat than unoccupied stands; for studies looking at amount of non-habitat, the unoccupied stands were used as the baseline, the hypothesis being that occupied stands are surrounded by *less* non-habitat than unoccupied stands.

The combined standardised effect-size was then calculated using multilevel linear mixed-effects models with the *rma.mv* function in the *metaphor* package in R [63], with study ID as random factor. Risk of publication bias was analysed using funnel plots (R package *metaphor*; 63) and test for asymmetry i.e. correlation test (coef_test R package clubSandwich) Robustness of results was quantified by calculating fail safe numbers [64]. To account for the study validity assessment, sensitivity analyses was attempted by running the analyses both with and without studies considered of medium risk of bias (no studies were considered of high risk). However, this was only possible for the analysis of presence. For the analyses of abundance and richness, a majority of studies were considered of medium risk of bias. For all except one study, the motivation for this was the lack of quantification of the difference in fragmentation between compared landscapes. This left too few studies to conduct meaningful sensitivity analyses by exclusion. However, since this type of bias should only risk false negatives, and the analyses was only used to identify a significant negative effect of landscapes but not quantify how big this effect was, we considered this risk of bias irrelevant for the analyses. No individual project was deemed to be overrepresented in the analyses, thus sensitivity analyses through exclusion of individual projects was not considered necessary.

Knowledge gaps were explored using heat maps cross tabulating the landscape factors, landscape scales, organism groups, outcome types, forest types, and countries studied.

## Results

**Fig. 2:**
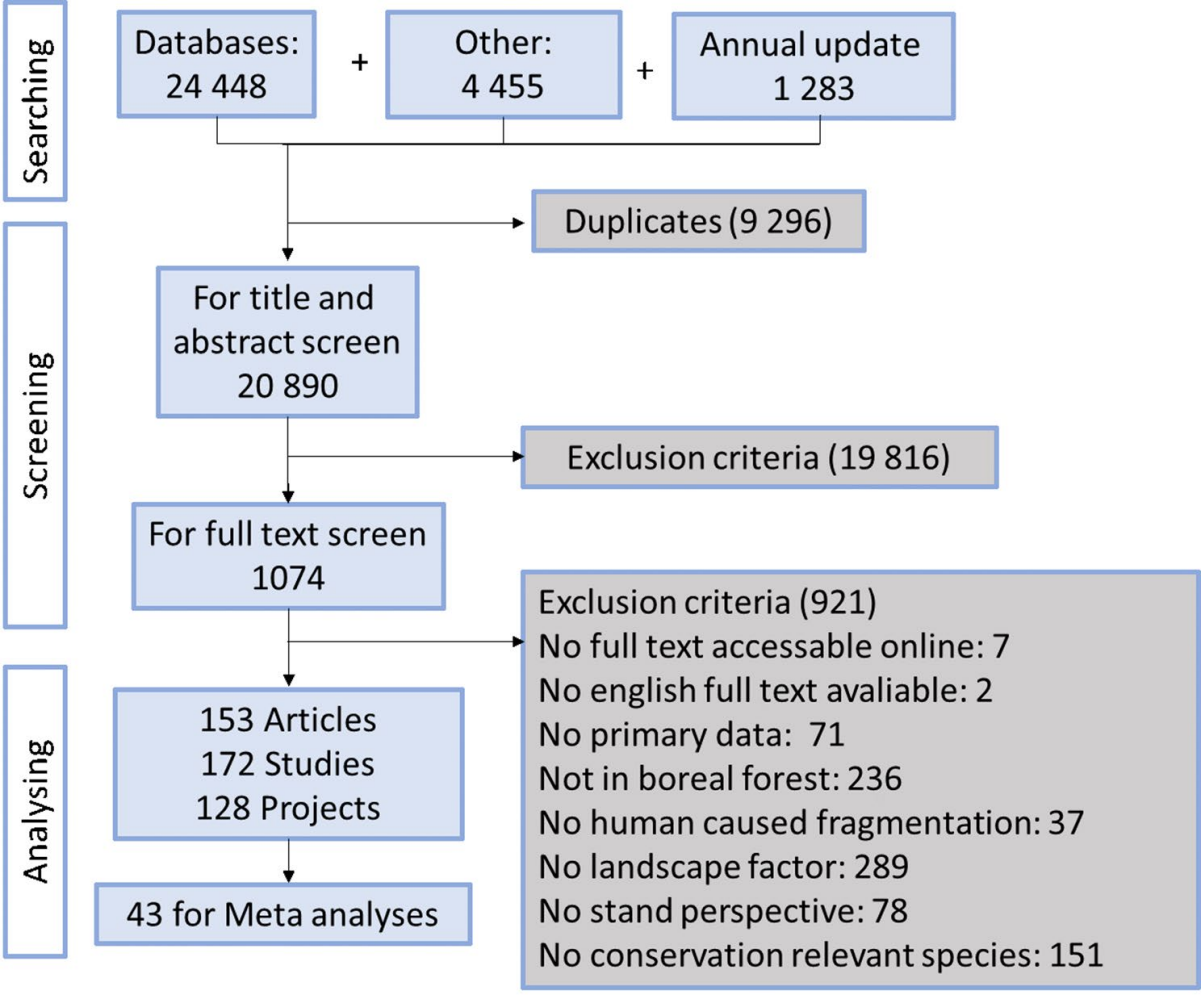
Flowchart illustrating the search, screening, and analyses process. Specifically, the number of articles identified in the search, removed due to being duplicates, that were exposed to title and abstract screening, excluded during this screening stage, exposed to full text screening, excluded at this stage, and the main reason for doing so. Lastly, the number of articles included in the review, how many studies those included, how many projects they originated from, and how many were suitable for meta-analyses

**Fig. 3:**
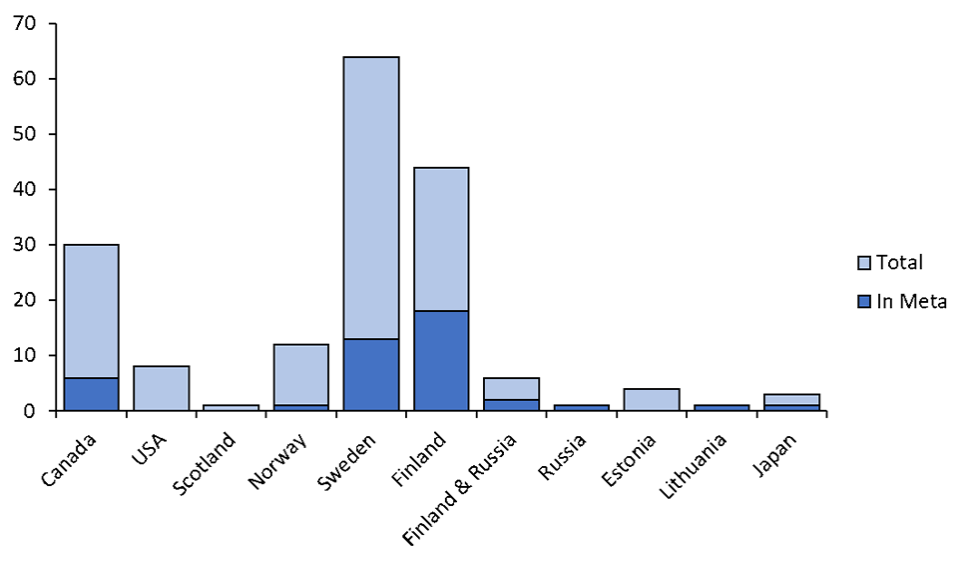
Bar graph showing the distribution across countries of the 172 included studies, and how many of those could be included in the meta-analyses

Our searches in databases plus specialist websites and other sources resulted in 30 186 articles (Fig. 2; Additional file 5). Of these, 9 296 were excluded based on being duplicates, leaving 20 890 titles and abstracts to screen. Just under 95% of these could be excluded during screening of titles and abstracts based on not meeting our inclusion criteria, leaving 1 074 papers for full text screening. During full text screening, 921 of these were excluded due to not meeting our inclusion criteria, or, in ten cases, that no full text or no English full text was available (Additional file 6). When looking at all criteria equally, the most common reasons for exclusion were that the studied species were not stated as conservation relevant, that none of the explanatory variables were linked to the surrounding landscape, or that the studies were conducted outside the boreal zone (or partly outside and the data from boreal stands could not be separated). When considering the exclusion criteria hierarchical, as depicted in Fig. 2 (i.e. a study conducted outside the Boreal forest that also lacked a landscape perspective is listed under Not in Boreal forest), the most common reason for exclusion was lack of landscape level variables. This left 153 articles, which upon further inspection were deemed to consist of 172 studies and stem from 128 research projects and 10 countries (Fig. 3). All included studies are listed in Additional file 4. Half of these were considered being of medium risk of bias, and the rest of low risk (Additional file 3), and no studies were excluded based on the validity assessment.

The landscape factors studied could be split into five categories: L1 Human vs. Natural fragmentation; L2 Categorical fragmentation intensity; L3 Amount of habitat, non-habitat, or its change over time; L4 Distance to habitat or non-habitat; and L5 Habitat configuration (spatial distribution of habitat; Fig. 1B). Only a few studies fell in category L1, and all originated from the same project. These studies looked at the difference between stands separated by wetland compared to stands fragmented by humans, and indicated a risk for extinction dept in stands fragmented by forestry. A large proportion of the studies fell into the L2 category. Most of these looked at fragmentation intensity in a rather general way, were stands were deemed as being in a more or a less fragmented landscape, but where no aspect of these landscapes was directly quantified. The other large group of studies were category L3 studies that directly quantified the amount of habitat, and/or the amount of non-habitat in the landscape surrounding the stand, with a smaller number of studies having quantified the historic amount of habitat. The third largest group of studies were category L4 that looked at distance to habitat or non-habitat. Lastly, some studies fell into category L5 having quantified habitat configuration, usually through an index accounting for number of habitat patches, their size, and their location. The variation in how this configuration index was done made these studies difficult to compare.

### Quantitative analyses

Forty-three studies had data that could be extracted and used in meta-analyses (Fig. 3; Additional file 7). This generated enough data to run three separate analyses for presence, abundance, and species richness, respectively, with sub analyses based on individual organism groups and/or landscape factors. Combinations of outcomes and landscape factors or organism groups not mentioned below were covered by too few studies (< 3) for meaningful meta-analyses.

**Fig. 4:**
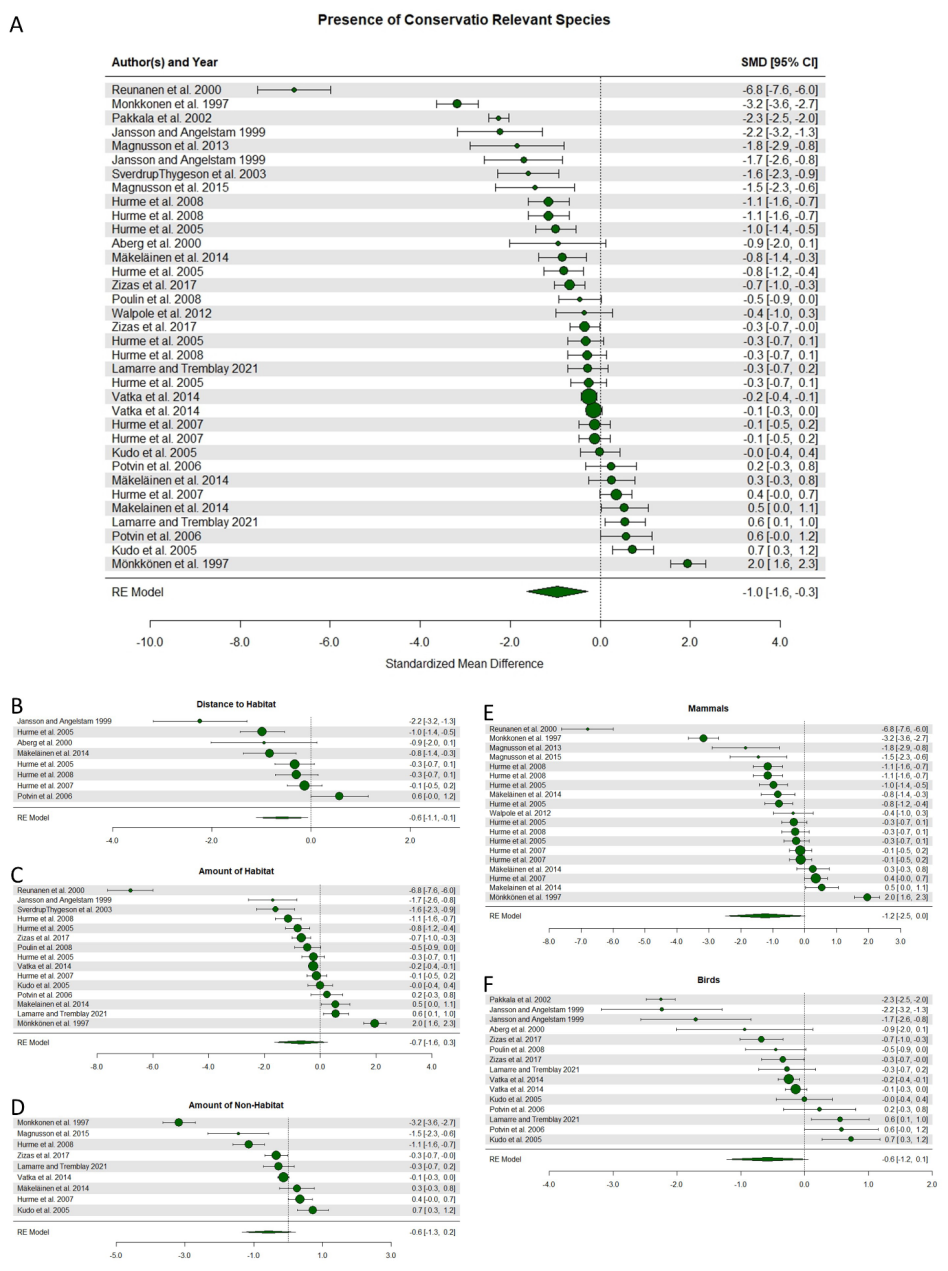
Forest plots illustrating the effect of fragmentation on presence of conservation relevant species. Negative values represent a negative effect of fragmentation; specifically, that unoccupied stands were found in more fragmented landscapes compared to occupied stands. Circles and numbers to the right indicate the mean effect size for each study. Whiskers and numbers in brackets indicate the 95% confidence interval. The size of the circle is proportional to the sample size. The rhombus at the bottom shows the combined effect size of the included studies. Panels include all studies (**A**), and subsets with studies looking at distance to habitat (**B**), amount of habitat (**C**), and amount of non-habitat in the surrounding landscape (**D**); at mammals (**E**), and at birds (**F**), respectively

The first analysis focused on presence of conservation relevant species and found that occupied stands were on average surrounded by a less fragmented landscape compared to unoccupied stands (Fig. 4A), i.e. there was support for an overall significant negative impact of fragmentation. This analysis included 34 comparisons with a standardized mean effect size ranging from − 6.8 to 2.0; for 16 comparisons, the effect size and entire confidence interval were consistent with a negative effect of landscape fragmentation (Fig. 4A). Absolut heterogeneity, σ^2^, was 2.299, and relative heterogeneity, I^2^, was 98.27%. The results remained significant after excluding studies considered of medium risk of bias, thus these studies were included in the final analyses. The test for funnel plot asymmetry was significant (Coef est. = -0.957, *p* = 0.012) and the failsafe number was three studies (Additional file 8, Supplementary Fig. 1A). When broken down further, the negative effect of landscape fragmentation on presence remained significant for studies looking at distance to habitat but not for studies looking at amount of habitat or non-habitat in the landscape; the effect was also nearly significant for the subgroups of studies looking at birds and mammals, respectively (Fig. 4B-F).

**Fig. 5:**
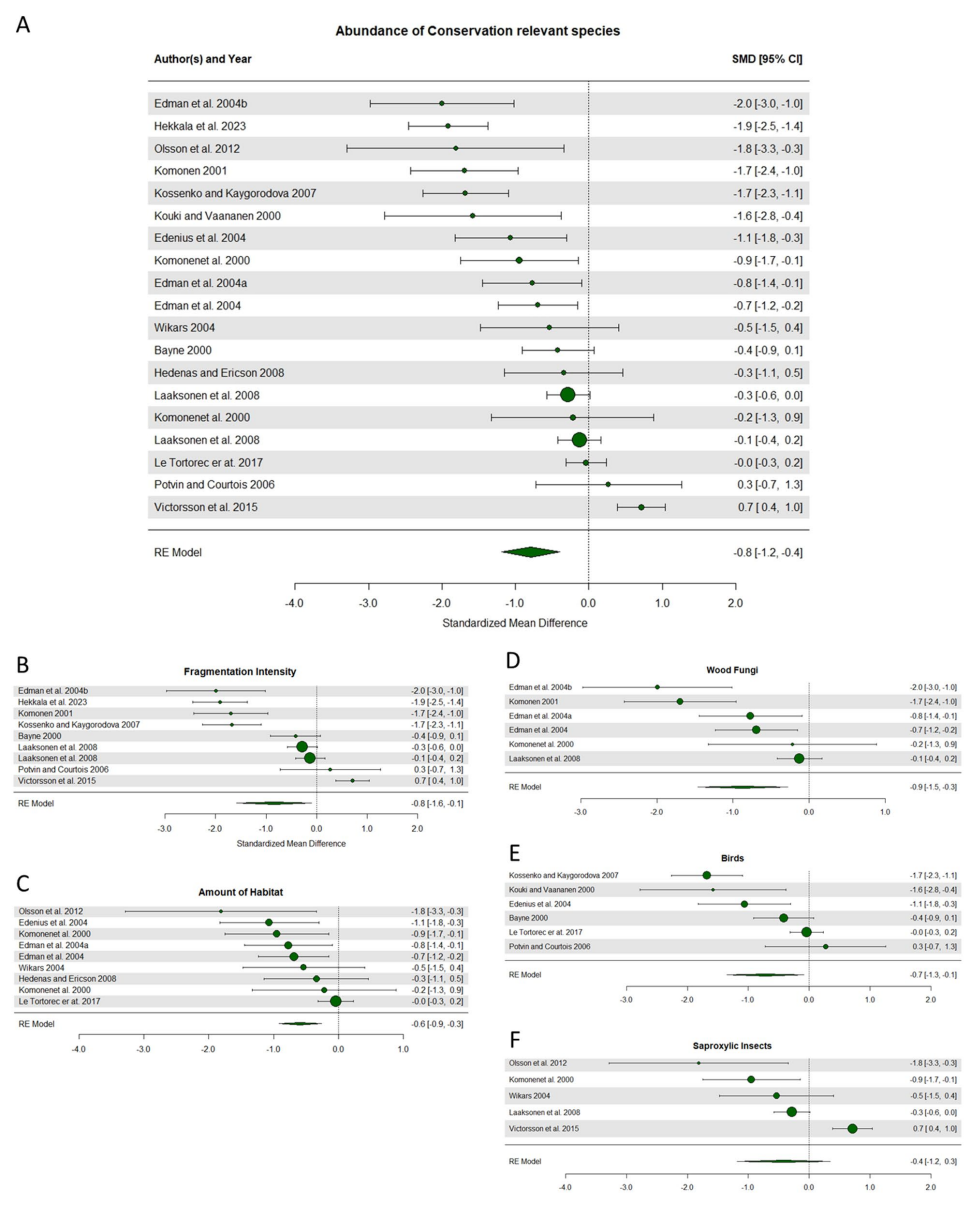
Forest plots illustrating the effect of fragmentation on abundance of conservation relevant species. Specifically, that abundance was lower in stands surrounded by a more fragmented landscape. Panels include all studies (**A**), and subsets with studies looking at categorical fragmentation intensity (**B**), amount of habitat (**C**); at wood fungi (**D**), saproxylic insects (**E**), and birds (**F**). For further explanation see Fig. 4

The second analysis focused on abundance and showed overall significant support for a negative effect of fragmentation on abundance (Fig. 5A). This analysis included 19 comparisons with a standardized mean effect size ranging from − 2.0 to 0.7; for 10 of the comparisons, the effect size and entire confidence interval were consistent with a negative effect of landscape fragmentation (Fig. 5A). Absolut heterogeneity, σ^2^, was 0.547, and relative heterogeneity, I^2^, was 88.43%. The test for funnel plot asymmetry was significant (Coef est. = X, p = X), and the failsafe number was 17 studies (Additional file 8, Supplementary Fig. 1B). When broken down further, a negative effect was confirmed both for studies looking at categorical fragmentation intensity and amount of habitat in the landscape (Fig. 5B and C), for studies of wood fungi (Fig. 5D) and birds (Fig. 5F), but the effect was not significant for saproxylic insects (Fig. 5E).

**Fig. 6:**
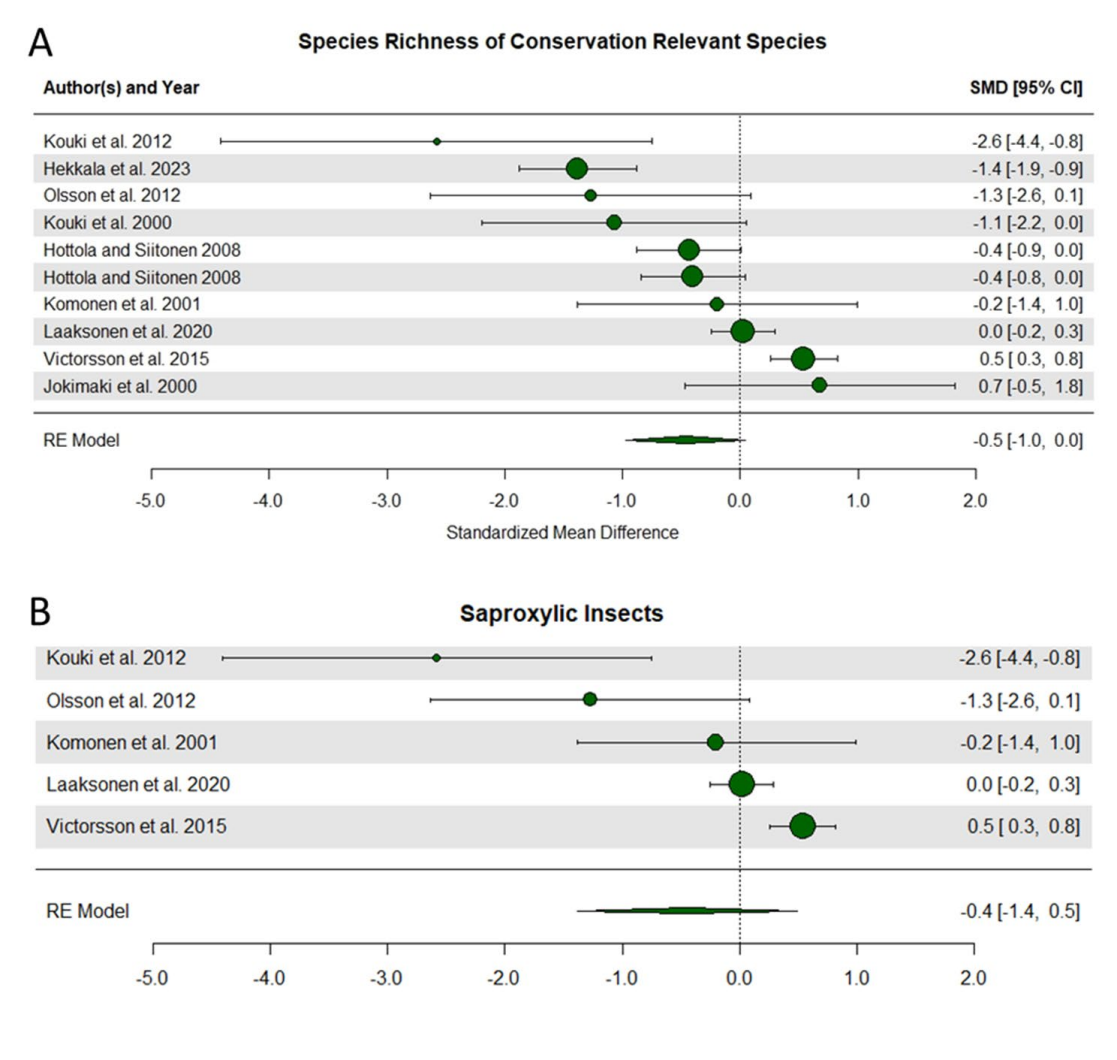
Forest plots illustrating the effect of fragmentation on species richness of conservation relevant species. Panels include all studies (**A**), and a subset with studies looking at saproxylic insects (**B**). For further explanation see Fig. 4

The third analysis focused on species richness and found a nearly significant support for a higher species richness in less fragmented landscapes (Fig. 6A). This analysis included 10 studies with a standardized mean effect size ranging from − 2.6 to 0.7; for 2 of the studies, the effect size and entire confidence interval were consistent with a negative effect of landscape fragmentation (Fig. 5A). Absolut heterogeneity, σ^2^, was 0.489, and relative heterogeneity, I^2^, was 87.68%. The test for funnel plot asymmetry was not significant (Coef est. = -0.465, *p* = 0.109; Additional file 8, Supplementary Fig. 1C). When broken down further, this trend was not true for studies of saproxylic insects, and no other sub analyses could be conducted (Fig. 6B).

### Narrative analyses

The variables, effect modifiers, and outcomes of all 172 studies were collected in a narrative table (Additional file 4). The evidence base they provide is summarised below.

### Landscape factors and organism groups

In general, there was strong support for an effect of the surrounding landscape on the occurrence of conservation relevant species in a given forest stand. The studies finding an effect of the landscape had mostly focused either on a scale of a ≤ 500 m radius or a 2–5 km radius, but all scales from < 250 m to regional scale were represented in the reviewed studies. The studies showing an effect of landscape had more commonly analysed the amount of habitat (historic or present) or non-habitat, compared to distance to habitat or categorical intensity of fragmentation.

In agreement with the meta-analyses, the strongest narrative support was found for a negative effect of a higher, categorical fragmentation intensity on abundance. When landscape variables were directly quantified (such as amount of habitat), the results were dependent on landscape scale used, how habitat or non-habitat were defined, and the species studied, resulting in a variable support for an overall effect.

### Mammals

In total, 29 of the identified studies covered the effect of the surrounding landscape on mammals. The majority of these articles focused on quantifying the differences in the landscape surrounding occupied *versus* unoccupied stands and mainly included studies from conifer-dominated forests in Canada, Sweden, and Finland. Most focused on the amount of habitat or non-habitat (often both) in landscapes ≤ 1 km in diameter. Distance to habitat or habitat configuration was also relatively commonly examined variables.

### Siberian flying squirrel

Eleven studies focused on Siberian flying squirrel (*Pteromys volans*). All of these looked at multiple landscape variables and on multiple landscape sizes. The majority of studies found a negative effect of fragmentation, with unoccupied stands being either further from habitat, surrounded by less habitat, or in a less connected landscape compared to occupied stands. However, there was no consistency among studies which of these three factors had an effect of squirrel presence, and most studies also found at least one of them to have no effect. The most ambiguous results were of the effect of amount of non-habitat in the landscape, where studies found either a positive, a negative, or no connection between squirrel occurrence and less non-habitat in the landscape.

### Grey-sided Vole

Grey-sided vole (*Craseomys rufocanus*) featured in six studies. The majority of these found a positive effect on presence or abundance of a more connected landscape with less non-habitat. However, some studies also found no effect, at least for some landscape variables, and one study even found a negative effect of a higher amount of habitat, depending on how this was defined.

### Other

Other mammals studied were American marten (*Martes Americana*; 3 studies), Woodland caribou (*Rangifer tarandus caribou*; 2 studies), bats (multiple species; 2 studies); and Snowshoe Hare (*Lepus americanus*), Red-backed Vole (*Clethrionomys gapperi*), Fisher (*Pekania pennanti*), Red squirrel (*Sciurus vulgaris*), Northern flying squirrel (*Glaucomys sabrinus*), Lynx (*Lynx lynx*), and shrews (multiple species) with 1 study each respectively. There was no clear trend among these, with fragmentation having a negative, positive or no effect depending on species, variables measured and/or how these variables were defined.

### Birds

In total, 55 studies examined birds and the effect of the surrounding landscape. Approximately half of these studied presence and the other half abundance, with a smaller number of studies focusing on species richness. The studies were conducted in Canada (19 studies), Finland [17], Sweden [8], USA [5], Estonia, Japan, and Russia (2 each), and Lithuania [1]. Compared to mammals, bird studies were conducted in a greater variety of stand types. The most common landscape size analysed was a radius of 500 m around the stand (21 studies) and the most common variable analysed was amount of habitat in the landscape (35 studies) or a combination of amount of habitat and amount of non-habitat (23 studies).

Overall, results were highly mixed for the effect of surrounding landscape on bird presence, abundance, and richness. This was the case for all landscape variables as well as all landscape sizes and all forest types. However, a substantial majority of the studies focusing on categorical fragmentation intensity (i.e. comparing landscape with “high” and “low” fragmentation respectively) found higher abundance of birds in less fragmented landscapes. The latter is consistent with the meta-analyses.

### Species and species groups

The bird studies spanned a large number of species, some reoccurring species and groups were grouse (14 studies; Capercaillie *Tetrao urogallus* most common), tits and allies (21 studies, Eurasian treecreeper *Certhia familiaris* the most common), and woodpeckers (18 studies of 6 different species). Most studies on Capercaillie found a positive effect of more habitat in the landscape on both presence and abundance (but one study found the opposite). The results for amount of non-habitat were inconclusive, potentially in part due to differences in how this was defined among studies. Almost all studies of treecreepers *Certhia sp.* found support for a negative effect of fragmentation. However, most of these studies included several landscape aspects, most of which were found to have no effect, and there was no specific factor dominating the landscape effect. For Woodpeckers, a majority of the studies found a negative effect of fragmentation on abundance, while most studies found no effect on presence.

### Saproxylic organisms

Deadwood dependent organisms featured in 77 studies conducted mainly in Fennoscandia and in coniferous forest. Most of these studies focused on abundance and species richness or a combination of both.

The trend of a negative effect of fragmentation in the surrounding landscape was strongest for species richness. This trend was even stronger when looking only at red-listed species. In terms of species composition, a large majority of studies looking at a 1 km radius landscape found an effect of landscape on species composition, while studies looking at fragmentation differences at the regional scale rarely found support for such an effect.

Looking at regional differences in fragmentation (usually forestry history), a majority of studies found an overall negative effect of fragmentation on abundance across species, but studies who broke this down further often found large variation in the effect on individual species. At smaller landscape scales, the variable effect on individual species, resulted in the lack of a general trend.

### Wood fungi

Of the 32 studies investigating wood fungi, the majority focused on regional difference in landscape fragmentation, defined by the present and/or historic amount of habitat, or on categorical fragmentation intensity based on forestry history.

The effect of landscape fragmentation on presence of wood fungi was highly variable among species, generating no overall pattern; the same was true for the effect of change in habitat amount over time. Likewise, the effect of fragmentation on species richness depended on which species/groups of species were included in the analyses. All these results were independent of stand forest type, and variable used to describe landscape fragmentation.

However, looking at individual species occurring in multiple studies, we highlight five redlisted, specialist species (*Fomitopsis rosea*, *Phlebia centrifuga*, *Phellinidium ferrugineofuscum*, *Phellopilus nigrolimitatus*, and *Amylocystis lapponica*) and two generalist species (*Trichaptum abietinum* and *Fomitopsis pinicola*). For these, the results were less ambiguous. For the redlisted species, almost all studies found a negative effect of fragmentation, especially in terms of a lower amount of habitat in the landscape. An effect of habitat configuration had less support. For the generalists, only about half of the studies found any effect of fragmentation. Distance to habitat and amount of non-habitat had rarely or never been investigate for any of these species.

### Insects

The 45 studies of saproxylic insects were conducted across all landscape scales, mostly focusing on amount of habitat, or categorical fragmentation intensity. Most of these studies focused on beetles. Very little support was found for an overall effect of fragmentation on presence of individual saproxylic insect species. However, half the studies looking at species richness of saproxylic insects found an effect of the fragmentation in the surrounding landscape.

### Lichens

Seventeen studies looked at lichens. These were almost exclusively conducted in Sweden, and mostly in coniferous forest stands. The majority focused on large-scale landscapes and regional differences in fragmentation, and most studies compared species richness (13 studies), with abundance also being relatively common (8 studies). The studies showed little support for an effect of landscape fragmentation on lichen species richness, presence, or abundance. Across all studies, there is a tendency that the lack of effect on abundance and presence could be an effect of individual species responding very differently to fragmentation. There was too little overlap between studies in included species to break this down further in a meaningful way.

### Other

Six studies focused on bryophytes. They demonstrated a fairly consistent positive effect of lower fragmentation intensity and a higher amount of historical habitat, both on species richness and abundance of bryophytes. These studies were all conducted in Sweden, but in different forest types.

Lastly there were three studies of vascular plants, one of non-saproxylic insects, and one of non-saproxylic fungi. There was no overall trend for the results of these.

### Landscape size, forest type, and stand vs. landscape

When looking at studies that included several landscape sizes (57 studies), the majority found that one or several sizes had a higher explanatory power for the occurrence of conservation relevant species. However, there was no support for a bell-shaped response, i.e., stronger explanatory power at intermediate scales; not when looking across nor within studies. Instead, support for an effect of landscape was similar for all scales up to 3500 m radius. When breaking this down per organism group, some tendencies appeared. For mammals and birds, explanatory power tended to decrease with an increasing landscape size, but the decrease was small and explanatory power remained high also for large landscapes. For wood fungi, the explanatory power of the landscape was equal up to a 2 km radius, and then declined somewhat, while remaining high for all scales. For saproxylic insects there was no trend between landscape size and explanatory power.

There were 80 studies that focused on more than one landscape factor. Habitat amount in combination with one more factor was the most common set up, frequently amount of non-habitat. These studies suggest that amount of habitat might be the more informative of the two, but there were also a number of studies that found effect for non-habitat but not amount of habitat.

Many of the studies had, in addition to landscape, also looked at the effect of one or more stand-level variables. Almost all studies that found an effect of landscape also found an effect of stand features. In addition, a majority of the studies that found no landscape effect did find an effect of one or more stand-level variables on occurrence of conservation relevant species.

### Knowledge gaps and potential bias

**Fig. 7:**
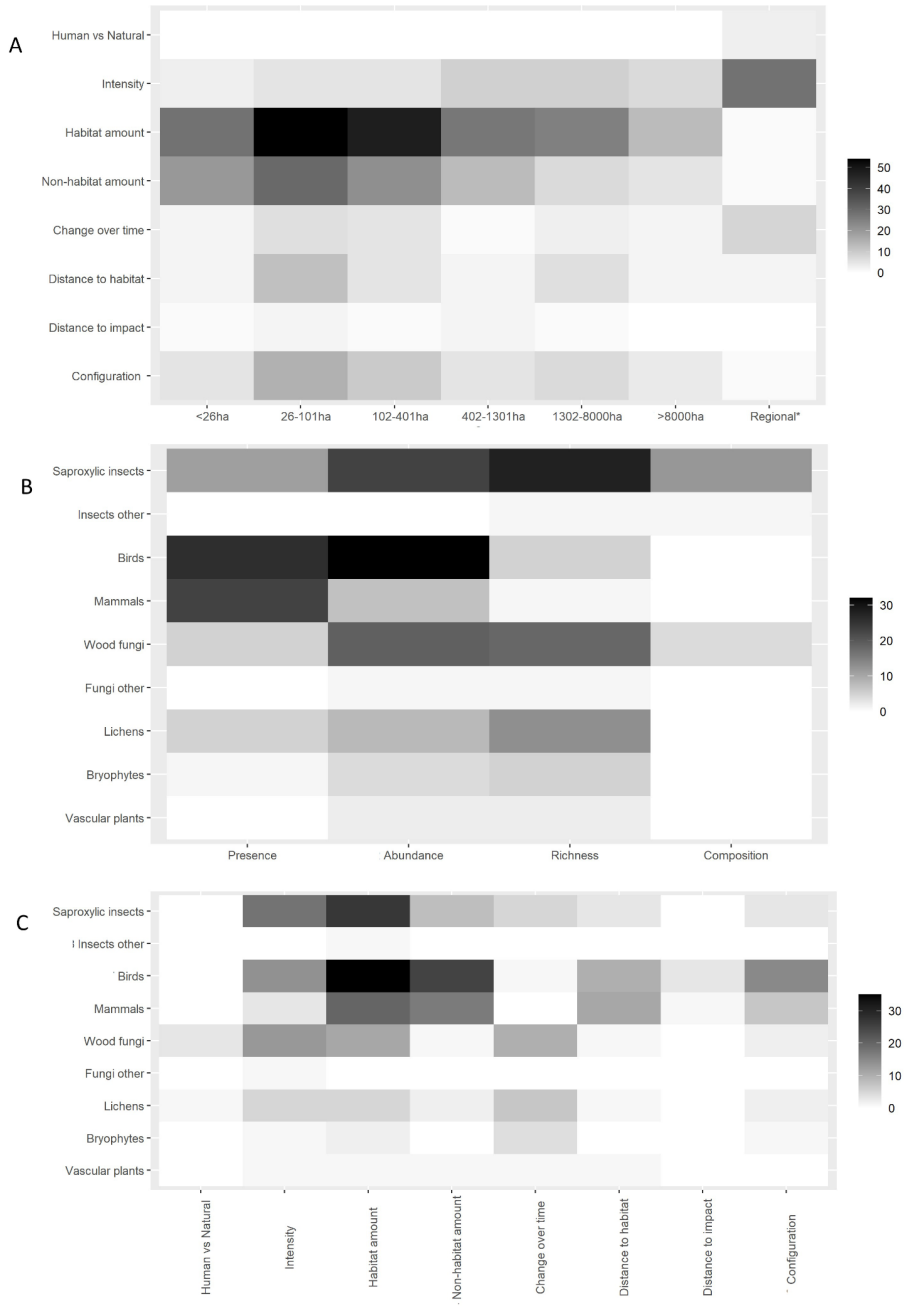
Heatmaps illustrating the number of studies that match different combinations of descriptors, and thus reflecting the evidence base, as well as knowledge gaps. The more studies, the darker the colour, the unit for the scales to the right in each panel is number of studies, note that this differs between the panels. Panel A shows which landscapes sizes were studied for which landscape factors. Panel B shows which outcome type was quantified for which organism group, and panel C which landscape factor was considered, when studying which organism group. “Regional” in panel A refers to studies comparing different parts of a country with differing fragmentation intensity and/or forestry history, but also included studies for which landscape size was unspecified. See main text for explanation of landscape factors. See further heat maps in Additional file 8

Four organism groups dominated the studies: mammals, birds, saproxylic insects, and wood fungi, all other groups were sparsely or not at all represented. Using heatmapping, we found that certain combinations of landscape scales, landscape factors, organism groups and outcomes were underrepresented in the published literature (Fig. 7; Additional file 8, Supplementary Fig. 2). For instance, studies of presence were the most common type for mammal studies but very uncommon for saproxylic organisms, and the opposite was true for species richness. Similarly, categorical fragmentation intensity was almost exclusively associated with a regional scale. Studies were overall skewed towards a landscape size of 500 to 1000 m radius. Studies of species richness were dominated by saproxylic insects. Studies of species composition were overall few. Studies of change in the landscape over time was also overall few. Geographically there was a dominance of studies from Sweden and Finland, a lower amount from Canada, and very few from Russia, despite the relative area of boreal forest in these countries (Fig. 3; and “Limitations” below). With one exception, the studies were published between 1995 and 2023, with a peak between 2004 and 2008.

The potential risk of bias identified in the study validity assessment was mainly that for studies comparing categorical fragmentation intensity, the intensity was rarely quantified, but rather deemed as higher or lower based on knowledge of regional forestry history. This may pose a risk of bias since the difference between high and low fragmentation intensity may differ between studies, which in turn could be correlated with whether an effect on fragmentation could be found. Another risk of bias we identified was that landscapes were frequently overlapping, or that the information of this was lacking, increasing the risk of pseudo replication. However, there were also studies where landscapes were so far apart that comparability could be questioned. A final reason for risk of bias was that the description and/or execution of the statistical analyses, primarily due to lack of details presented.

Regarding the studies included in the meta-analyses, and for studies of presence, only a smaller number had minor concerns relating to the reporting of landscape overlap and/or statistical analyses. Among the studies of abundance, about two thirds were deemed of medium risk of bias because the difference in fragmentation intensity was not quantified, but this could only result in a bias towards non-significance, and the sub analyses with only such studies remained significant. For studies of species richness, the proportion of studies based on fragmentation intensity not quantified was even larger and this in combination with the relatively few studies in this analysis might be vulnerable towards not detecting a negative effect.

### Review limitations

The main limitation of this review lies in its attempt to combine and summarise a very heterogenous set of studies. However, despite this heterogeneity, two of our three meta-analyses found a significant signal for a negative effect of fragmentation.

Another potential limitation is that the evidence base for this review was built on, did differ among organism groups. The criteria used to define conservation relevant species contributed to this. Surprisingly, many studies were excluded based on not stating the conservation-relevance of their study species. For instance, very few studies of lichens and bats made such statements, and for other groups studies of the same species could fall either side of inclusion due to the lack of explicit statement of the species as conservation relevant. However, we withhold that using the authors’ definition on conservation relevant was the most objective way to include studies in the review.

In addition, we acknowledge that the review authors and stakeholders involved were (with one exception) Swedish. A relevant question is whether this is the reason for a majority of studies originating from Fennoscandian countries. To mitigate potential Swedish influence, the benchmark list included papers from a number of countries, thereby attempting to reduce the risk of using search terms biased towards Fennoscandian studies. This approach was supported by a large variety of origin of the studies passing title and abstract screening. Instead, the main reason for the bias towards Fennoscandian studies was the criterion focusing on stand-level research. This criterion excluded a larger proportion of the North American studies since these often focused on the landscape or species distribution level and/or involved species unsuitable for a stand-level analyses (such as large mammals). We speculate that the reason for this difference reflects a combination of research tradition and the more fragmented nature of the Fennoscandian forest, leaning itself better towards a stand-level focus. Studies from Russia were also few. This could be due to such studies being published in Russian and thus not accessible to us, or that studies matching the inclusion criteria are rarely conducted in Russia. The previous experience of the review authors in combination with the descriptive nature of most Russian studies appearing in the search results, makes the review authors lean towards the latter, and that additional search through Russian sources was not motivated.

Truly meaningful forest type categorisation of stands and landscapes was challenging, in part due to lack of detail in the descriptions in the articles, but also due to the large variation across the vast geographic region the review covered. This somewhat limits the potential for conservation managers to address questions related to particular forest types.

Another limiting factor was that many studies relied on unquantified fragmentation differences among landscapes. Frequently, general statements about a longer history of forest management were used to split regions into more and less fragmented. This makes the compilation of data difficult since there is no way to account for the magnitude of fragmentation difference among studies. This contributed to that, while our analyses show that there is an effect of the landscape, we could not numerically quantify the extent of this effect.

The results of the asymmetry tests suggested a risk of bias towards large studies with significant results among those included in the meta-analyses. However, another explanation resulting in the same asymmetry is large heterogeneity among studies. We find the latter reason likely due to the substantial amount of heterogeneity, but a publication bias cannot be excluded. The main limiting factor for including more studies in the meta-analyses was the tendency to only report modelling output and related p-values, rather than actual number of observations or variable ranges. In addition, such models tended to include multiple variables on multiple scales, making the interpretation and summary in the narrative table format difficult.

### Review conclusions

Our systematic review of published articles clearly shows that there is a negative effect of fragmentation of the surrounding landscape on the occurrence of conservation relevant species. The strongest support was found for a negative effect of a lower amount of habitat in the matrix on abundance. The extent and details of this negative effect did differ among organism groups as expected. However, there was little support for a stronger effect for species with poor dispersal; abundance of birds was as affected as abundance of wood fungi.

While amount of habitat had a clear effect, many studies found support for an effect of other measures of fragmentation, such as distance to habitat, fragmentation intensity, and/or amount of non-habitat. However, as different measures of fragmentation were rarely compared, we cannot draw any conclusion which measure provide the best information of fragmentation effects. This is expected due to the large differences in ecology and life history strategies among studied species, and ideally choice of fragmentation measure should be based on a clear hypothesis on how fragmentation is manifested for the species/species group.

Contrary to our expectations, there was no support for a bell-shaped relationship between landscape size assessed and explanatory power. This suggests that there is no single scale that best explain the impact of fragmentation, and hence it is difficult to recommend a scale at which to account for landscapes in forest management. On the contrary, there was plenty of evidence for effects relating to landscape radii from 250 m up to several kilometres and even on a regional scale. The importance of landscape fragmentation seemingly did not differ between forest types, and regardless of forest type, most studies found effects both of landscape and stand-level features on the occurrence of conservation-relevant species, underlining that both scales are important to consider and account for.

### Implications for policy and management

To preserve and benefit conservation relevant boreal forest species, focusing on stand-level features is not enough. Our review show plenty of support for that both the amount of habitat and how far away it is located affects stand-level species occurrence; that this can be the case on all scales from a few hectares to entire regions; and that this was the case across forest types and organism groups. These results emphasize the negative effects of the practice of intensive forestry and associated landscape transformation that has occurred during the last century. Instead, we urge policy makers and forest management to see this review as additional support for the need to shift towards a forestry that preserves remaining old forest and targets restoration actions to improve landscape connectivity where this has been lost. This calls for spatial landscape planning based on comprehensive geographical information that support positioning of future protected area and forest restoration [65, 66]. However, although we show that landscape fragmentation is an important factor to consider, our review should not be interpreted as if these are generally more important than stand level factors. How to prioritize between focusing on single, isolated high-quality stands or on lower quality stands in less fragmented landscapes remains an issue to be considered case by case.

### Implications for research

This review points out several areas where more research is needed. We find it troubling that so few of the studies focused on a change in the landscape as well as in occurrence over time. Such studies are very important since the full effects of fragmentation might not be visible for years or decades, and seemingly viable populations risks representing extinction dept. One way forward for this is to utilise the studies included herein and resurvey both landscapes and populations in the same areas years later.

To truly quantify the negative effect of landscape fragmentation, we urge future studies to directly quantify fragmentation, rather than using a vague division in more and less fragmented landscapes. How much habitat remain in the respective landscapes? How far away is it situated? And how long ago did fragmentation take place? Also from this angle, a way forward would be returning to conducted studies and quantify landscape fragmentation and redo analyses. This could be done for instance through utilising landcover data, satellite imaging, and/or machine learning methods (see for instance 68), and would further the understanding of the degree of negative effect and crucial aspects such as threshold values. Further, we encourage more publications to, in addition to models, also include basic statistics such as means and standard deviation, to make results more accessible and comparable. We also warrant authors to be clearer about whether and why their study species can be considered conservation-relevant, not the least to make the studies comparable and accessible to readers unfamiliar with the species or group.

Overall, we urge for more studies with a landscape perspective. A promising way forward for studying the effect of the surrounding landscape is remote sensing technology. Especially since a recent review on the topic found that boreal forest was underrepresented in such studies [53].

## Electronic supplementary material

Below is the link to the electronic supplementary material.

**Additional file 1**. Summary Stake holder meetings.

**Additional file 2**. The filled in ROSES reporting standards form for systematic reviews.

**Additional file 3**. Results of the study validity assessment (critical appraisal) and explanations of its criteria. Table of the criteria and how and why each article was categorised as being of high, medium, or low risk of bias.

**Additional file 4**. Narrative table including all 172 studies included in this review.

**Additional file 5**. Specification search results.

**Additional file 6**. Reasons for exclusion for all articles excluded during full text screening.

**Additional file 7**. Data used for the meta-analyses.

**Additional file 8**. Supplementary figures.

## Data Availability

Not applicable.
