# Supplementary material for: To what extent does surrounding landscape explain stand-level occurrence of conservation-relevant species in fragmented boreal and hemi-boreal forest? – a systematic review
Source: Environ Evid. 2024 Aug 12;13:19. doi: 10.1186/s13750-024-00346-1 (PMC11378823; doi:10.1186/s13750-024-00346-1)
Supplement: Supplementary file 1 — Additional file 1. Summary Stake holder meetings. [file 13750_2024_346_MOESM1_ESM.docx]

First stakeholder meeting, December 14, 2021

Participants included six representatives from County Administrative Boards in the boreal region of Sweden, one representative each from the Swedish Forest Agency and the Swedish Environmental Protection Agency, and three representatives from the major forest companies in Sweden.

The meeting started with a presentation of the participants and their role on forest conservation work. We then presented the Systematic Review question and how we intend to perform the review. The participants were then requested to reflect and comment on the following questions.

1. Is it your impression that the surrounding landscape influence species occurrences?
2. Do you currently consider the landscape scale in your work?
3. What types of landscape factors do you consider most relevant?
4. Which landscape size is most relevant for you?
5. Which species groups is most relevant for you?

Short summary of the discussion for each question

Q1/2. Pressure exists to consider the landscape and evaluate it in terms of green infrastructure. The Swedish Environmental Protection Agency frequently discusses the concept of “value tracts”, which typically encompass larger areas with multiple value cores, and are commonly utilized in landscape planning, particularly by county governments. The significance of the landscape is emphasized, especially in the context of conservation efforts for endangered insects. However, there are occasional remarks such as “you have the forest you have” and “valuable areas lie where they are”, which somewhat restrict the integration of landscape considerations in planning and prioritization. Forestry companies, on the other hand, emphasize that for areas in the "grey zone" between nature conservation and forest production zones, the location can significantly affect its status, for instance, in relation to adjacent reserved areas, considering the surrounding landscapes in the process.

Q3. How the effect of fragmentation and surrounding landscape differs between forest types was highlighted as important. Another important aspect that was highlighted was the relationship between amount of forest and distance/isolation, for example, making larger areas or clustering several smaller stands with high nature values ​​so that the distance to the next stand/area/cluster can be longer before it becomes functionally isolated ( i.e. before there are negative effects on diversity)? Is there any amount of forest in the landscape that cancel the negative effects of fragmentation?

Another factor that came up as important is what the "non-habitat" between the fragments consists of. Dispersal barriers? Clearcut? Dense understory? Young forest? What type of forestry has been carried out? Wetlands? This is to clarify what constitutes an acceptable fragmentation and what is a "hostile matrix".

Several times the time aspect was brought up. Is it really more important what the forest looks like now for the current diversity in a stand? Or how it once looked? If so when? And how does this relate to extinction debt. It was also pointed out during the meeting that studies that look at changes over time are probably unusual, but that it would be important for us to point out that such studies are needed and that the conditions for them have improved considerably with access to different types of historical data and analysis methods.

A specific, time-related factor that was highlighted was if there is an effect of how the forest has been owned and how this has changed over time.

Q4. When discussing what is a relevant size of a landscape, a recurring comment was that it would be strange if managers would define this. Rather it is research itself that should determine what size is relevant, not the other way around. That is, the stakeholders suggest grouping the studies based on the size of the studied landscape and see how this affects the results. Which scale has the highest level of explanation? Is there any largest landscape larger than which the results no longer change.

There seemed to be some consensus that an area smaller than 1km2 (i.e., 100ha) is not to be considered a landscape. At the same time, there is an optimal ecological scale that research can produce, but it may differ from what is a practically possible implementation scale. The implementation scale, in turn, partly depends on what you have at your disposal, for example most nature conservation in Sweden is based on counties and county borders. In Norrbotten County, they are apparently talking at the 10,000 ha level (i.e. 10x10km). The State forest company’s Ecoparks and corresponding areas of other forest companies are usually in the order of 4000-5000 ha (although some are considerably larger) - but within such an area, it may be that only 50% is forest with natural values. One meeting participant is co-author of a study on gray-sided vole which found that 5x5km (2,500 ha) had a high degree of explanation. If more studies support that this is a relevant scale (i.e. a few square kilometers), then it is possible to do things within a forestry company, for example - which makes it easier to implement.

Related to this, the question came up if the county level is really the right level? Shouldn't it be decided from a higher place which areas of the boreal forest must be protected based on the distribution of red-listed species?

Q5. This topic was not discussed at length. Only a few species were mentioned specifically. On a direct question, it was also agreed that woodpeckers are interesting. Species with poorer dispersal ability are likely to be the ones most affected by fragmentation and are thus interesting.

Great interest was also expressed in whether the trends/effects are similar for different species. And by extension: Do indicator species work? That is, do they follow the same pattern as the red-listed ones? And do red-listed species follow the same trends so that one can identify a few red-listed species to use as umbrella species?

Second stakeholder meeting, 6 February 2024

Participants included nine representatives from County Administrative Boards in the boreal region of Sweden, two representative each from the Swedish Forest Agency and the Swedish Environmental Protection Agency, and five representatives from the major forest companies in Sweden.

The meeting started with a presentation of the participants and their role on forest conservation work. We then presented the Systematic Review process, methods, results, and conclusions. The participants were then requested to reflect and comment on the following questions.

1. Was the identified evidence base and the results of the meta-analyses relevant and understandable?
2. What were the most important parts missing? What are the most important knowledge gaps to fill in order to make the research more relevant for conservation practitioners?

Short summary of the discussion for each question

Q1. The stakeholders overall deemed the results as relevant and understandable. The compiled picture the review paints adds support to that landscape focus is required and provides welcome “weight” in these argumentations. In addition, the narrative table will act as a welcome and valuable resource for further reading, such as regarding specific organism groups of interest. Thus, the overall opinion was that the review will be a welcome and well used source in future discussions on management and restoration.

Q2. The main drawback with the review from a stakeholder perspective is that it just concludes that landscape fragmentation has a negative effect, not how large this effect is or at what point a landscape start being fragmented enough to have such negative effects. The discussion identified three knowledge gaps that the stakeholders suggest focusing on: 1) is there a threshold value for how fragmented a landscape can be before negative effects emerges? If so, how is this defined? By the amount of remaining habitat? It location? What it consists of? 2) how much does the effect change depending on the composition of the “non-habitat” in between habitat patches? For instance, I the negative effect larger if the habitat patches are separated by plantation forest, compared to clear cuts, compared to agricultural land. 3) What are the effects over time? How does populations change with time since fragmentation? How do they relate to historic amount of habitat and for how long does such patterns remain?
