# Supplementary material for: To what extent does surrounding landscape explain stand-level occurrence of conservation-relevant species in fragmented boreal and hemi-boreal forest? – a systematic review
Source: Environ Evid. 2024 Aug 12;13:19. doi: 10.1186/s13750-024-00346-1 (PMC11378823; doi:10.1186/s13750-024-00346-1)
Supplement: Supplementary file 8 — Additional file 8. Supplementary figures. [file 13750_2024_346_MOESM8_ESM.docx]

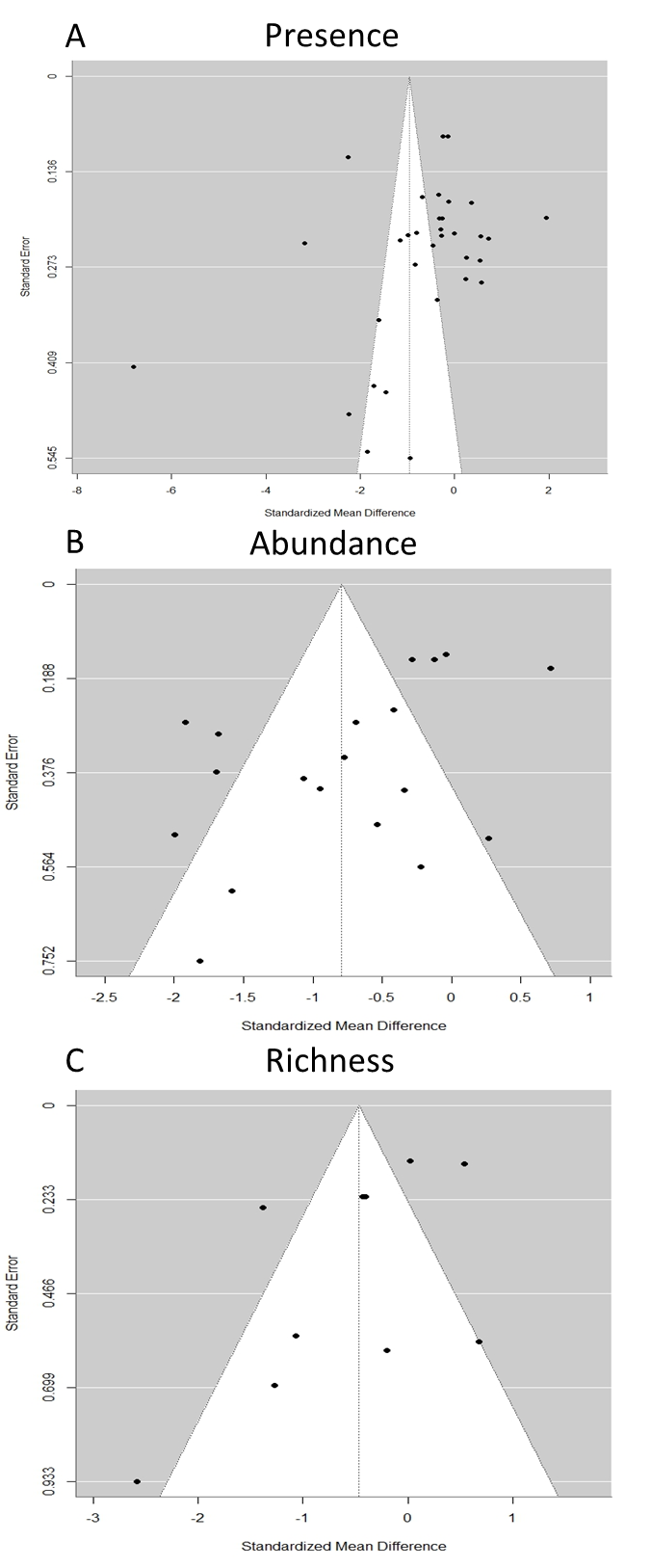
**Additional file 7.** Supplementary figures.

Supplementary Figure 1. Funnelplots of the symmetry of studies included in the meta-analyses of (A) presence, (B) abundance, and (C) richness of conservation relevant species. See main text for results of associated Egger’s tests.


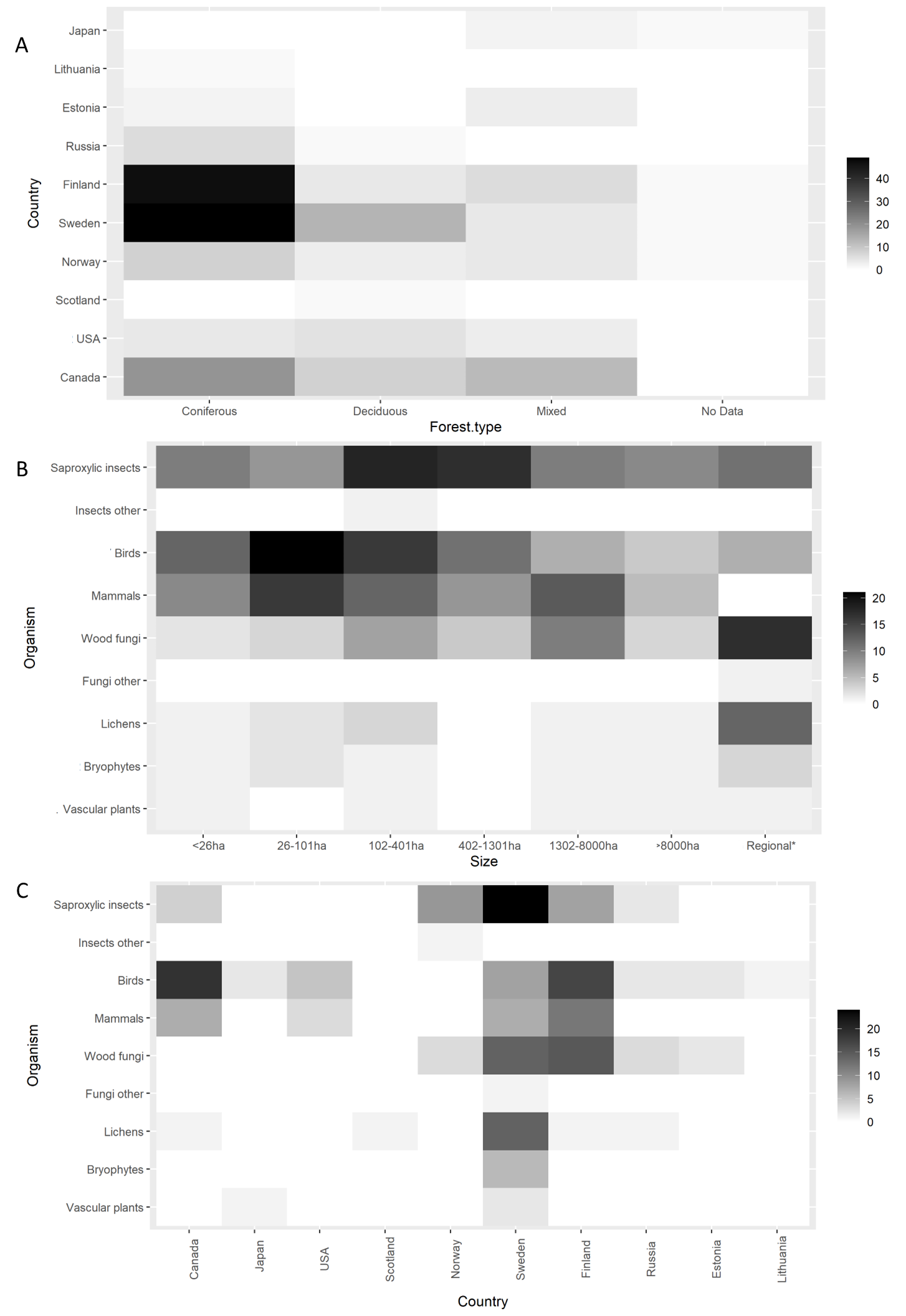


Supplementary Figure 2. Heatmaps illustrating the number of studies that match different combinations of descriptors, and thus the evidence base, as well as knowledge gaps. Complementary to Figure 7 in the main text. Panel A shows which forest types were studied in which countries. Panel B shows which organism group was studies at what landscape scale. Panel C shows which country studies of different organism groups originated from. Regional in panel B refers to studies comparing different parts of a country with differing fragmentation intensity and/or forestry history, but also includes studies for which landscape size was unspecified. The more studies, the darker the colour. Scale to the right indicates which nuance corresponds to how many studies, note that this differs between the panels.
